# Prediction and suppression of HIFU-induced vessel rupture using passive cavitation detection in an *ex vivo* model

**DOI:** 10.1186/2050-5736-2-14

**Published:** 2014-09-08

**Authors:** Cameron L Hoerig, Joseph C Serrone, Mark T Burgess, Mario Zuccarello, T Douglas Mast

**Affiliations:** 1 University of Cincinnati, Cincinnati, OH 45267-0586, USA

**Keywords:** High-intensity focused ultrasound, Vessel occlusion, Passive cavitation detection, Rupture suppression

## Abstract

**Background:**

Occlusion of blood vessels using high-intensity focused ultrasound (HIFU) is a potential treatment for arteriovenous malformations and other neurovascular disorders. However, attempting HIFU-induced vessel occlusion can also cause vessel rupture, resulting in hemorrhage. Possible rupture mechanisms include mechanical effects of acoustic cavitation and heating of the vessel wall.

**Methods:**

HIFU exposures were performed on 18 *ex vivo* porcine femoral arteries with simultaneous passive cavitation detection. Vessels were insonified by a 3.3-MHz focused source with spatial-peak, temporal-peak focal intensity of 15,690–24,430 W/cm^2^ (peak negative-pressure range 10.92–12.52 MPa) and a 50% duty cycle for durations up to 5 min. Time-dependent acoustic emissions were recorded by an unfocused passive cavitation detector and quantified within low-frequency (10–30 kHz), broadband (0.3–1.1 MHz), and subharmonic (1.65 MHz) bands. Vessel rupture was detected by inline metering of saline flow, recorded throughout each treatment. Recorded emissions were grouped into ‘pre-rupture’ (0–10 s prior to measured point of vessel rupture) and ‘intact-vessel’ (>10 s prior to measured point of vessel rupture) emissions. Receiver operating characteristic curve analysis was used to assess the ability of emissions within each frequency band to predict vessel rupture.

Based on these measurements associating acoustic emissions with vessel rupture, a real-time feedback control module was implemented to monitor acoustic emissions during HIFU treatment and adjust the ultrasound intensity, with the goal of maximizing acoustic power delivered to the vessel while avoiding rupture. This feedback control approach was tested on 10 paired HIFU exposures of porcine femoral and subclavian arteries, in which the focal intensity was stepwise increased from 9,117 W/cm^2^ spatial-peak temporal-peak (SPTP) to a maximum of 21,980 W/cm^2^, with power modulated based on the measured subharmonic emission amplitude. Time to rupture was compared between these feedback-controlled trials and paired controller-inactive trials using a paired Wilcoxon signed-rank test.

**Results:**

Subharmonic emissions were found to be the most predictive of vessel rupture (areas under the receiver operating characteristic curve (AUROC) = 0.757, *p* < 10^-16^) compared to low-frequency (AUROC = 0.657, *p* < 10^-11^) and broadband (AUROC = 0.729, *p* < 10^-16^) emissions. An independent-sample *t* test comparing pre-rupture to intact-vessel emissions revealed a statistically significant difference between the two groups for broadband and subharmonic emissions (*p* < 10^-3^), but not for low-frequency emissions (*p* = 0.058).

In a one-sided paired Wilcoxon signed-rank test, activation of the control module was shown to increase the time to vessel rupture (*T*_-_ = 8, *p* = 0.0244, *N* = 10). In one-sided paired *t* tests, activation of the control module was shown to cause no significant difference in time-averaged focal intensity (*t* = 0.362, *p* = 0.363, *N* = 10), but was shown to cause delivery of significantly greater total acoustic energy (*t* = 2.037, *p* = 0.0361, *N* = 10).

**Conclusions:**

These results suggest that acoustic cavitation plays an important role in HIFU-induced vessel rupture. In HIFU treatments for vessel occlusion, passive monitoring of acoustic emissions may be useful in avoiding hemorrhage due to vessel rupture, as shown in the rupture suppression experiments.

## Introduction

High-intensity focused ultrasound (HIFU) is a therapeutic modality with multiple clinical applications [1], many of which involve tissue ablation for treating of neoplastic pathology [2]. Neurosurgical applications of HIFU have been investigated since the early work of the Fry brothers [3,4]. HIFU has also been shown to occlude *in vivo* blood vessels in animal models [5-16]. This could have neurosurgical applications in pre-operative occlusion of feeding arteries to arteriovenous malformations (AVM) or large tumors, as is already performed with endovascular therapy.

While previous *in vivo* experiments have shown that HIFU-induced vessel occlusion is feasible, they have also demonstrated a risk of hemorrhage. In a recent paper [16] summarizing results from multiple studies of HIFU-induced vessel occlusion [5-14,17-20], vessel rupture was found to occur in some studies for 13% to 80% of the vessels targeted for occlusion [6-9,14]. Using a 1.49-MHz transducer with focal intensities 4,400–8,800 W/cm^2^, Hynynen et al. [6] occluded 19/27 sonicated rabbit femoral arteries and veins (diameter 1–1.3 mm), but found increased hemorrhage rates at higher intensities (hemorrhage in 0/3 vessels treated at 4,400–5,300 W/cm^2^, 4/19 vessels treated at 5,800–6,300 W/cm^2^, and 1/4 vessels treated at 7,300–8,800 W/cm^2^). In another study using a 1.5-MHz transducer with focal intensities of 2,800–6,500 W/cm^2^ to noninvasively occlude segmental rabbit renal arteries (0.6-mm diameter), the same group identified contrast extravasation on X-ray angiography in 2/9 vessels [7]. Rivens et al., using a 1.7-MHz transducer at a focal intensity of 4,660 W/cm^2^, occluded 4/10 rat femoral arteries and veins (0.5–1.5 mm), but detected hemorrhage by magnetic resonance angiography in 8/10 cases [8]. The same group, using a 1.7-MHz transducer to occlude rat femoral arteries and veins at intensities of 1,000–4,660 W/cm^2^, occluded 100% of vessels at spatial peak intensities of 1,690 W/cm^2^ without hemorrhage, but again demonstrated hemorrhage in both vessels treated at 4,660 W/cm^2^[9]. More recently, Ichihara, using a higher frequency transducer (2.2 MHz) with focal intensity of 4,000 W/cm^2^ for occlusion of segmental renal arteries in rabbits, found hemorrhage by contrast ultrasound on 3/8 cases [14].

Thus, although vessel occlusion has been demonstrated with HIFU for *in vivo* animal models, hemorrhage can occur under many exposure conditions within the range of interest investigated for occlusion. In a clinical setting, hemorrhage within the central nervous system, also a concern for ultrasound-enhanced thrombolysis [21] and therapeutic opening of the blood–brain barrier [22], can lead to catastrophic neurologic damage. Therefore, causes and possible mitigation for hemorrhage risks must be addressed prior to clinical application of HIFU for blood vessel occlusion in the central nervous system.

The mechanisms of vessel rupture after application of HIFU are presently unclear. One possible mechanism is supratherapeutic heating of the vessel wall. Hynynen et al. [7] reported that hemorrhage occurred after retreatment of previously treated constricted vessels, leading them to conclude that HIFU-generated heat was not dissipated due to reduced convection, causing supratherapeutic heating and vessel rupture. In a study evaluating the effects of argon lasers on rat carotid arteries, Martinot et al. [23] noticed severe tissue disorganization and perforation at temperatures greater than 100°C. Mechanical damage can also be caused by local tissue boiling, even at short duration, with radiation force playing a concomitant role [24]. Heating to 100°C, which causes tissue vaporization (boiling), has also been shown to cause mechanical damage such as cracking in thermally ablated tissue [25]. Tissue vaporization can be detected by low-frequency emissions [25-27].

Another possible mechanism of HIFU-induced vessel hemorrhage is the mechanical effects of cavitation. Inertial cavitation, or the violent collapse of microbubbles in an ultrasound field, is known to cause high-velocity microjets as well as concentrated high pressure and temperature, resulting in substantial mechanical damage to tissue under some conditions [28-31]. Tissue homogenization by the mechanical effects of inertial cavitation, a process known as histotripsy, has been applied to thrombolysis [32] and perforation of the atrial septum [33]. In addition, stable cavitation, or the sustained large-amplitude oscillation of microbubbles in an ultrasound field, is believed to cause mechanical effects due to microstreaming [34]. Bioeffects associated with stable cavitation are believed to include ultrasound-enhanced thrombolysis [35,36] as well as enhanced permeability of blood vessels [37], opening of the blood–brain barrier [38], and increased transdermal drug delivery with sonophoresis [39]. Mechanical effects of cavitation may also combine with those induced by vessel wall heating and radiation force. Acoustic cavitation activity can be detected from passively recorded acoustic emissions, with stable cavitation signified by subharmonic and ultraharmonic (i.e., integer multiples of subharmonic frequencies) emissions and inertial cavitation signified by broadband acoustic emissions associated with bubble collapse [40,41]. Passive cavitation detection has been previously employed in multiple studies of ultrasound ablation [25,42-45].

The first part of the study reported here, testing prediction of vessel rupture, employed application of HIFU (3.3 MHz) to *ex vivo* porcine superficial femoral arteries with passive cavitation detection while monitoring for rupture. This portion of the study tested the hypothesis that passively detected emissions during HIFU application to *ex vivo* vessels can predict impending vessel rupture, with the goal of providing insight into mechanisms of HIFU-induced vessel rupture.

If vessel rupture could be predicted with sufficient accuracy from measured emissions, passive cavitation detection could be used during vessel occlusion with HIFU in a clinical setting. Feedback based on measured acoustic emissions could vary sonication amplitudes, potentially allowing greater HIFU energy delivery while avoiding activity predictive of vessel rupture. A similar approach was recently employed for the application of blood–brain barrier disruption, using feedback control to modulate the amplitude of focused ultrasound to achieve stable cavitation while avoiding inertial cavitation [46]. For the application of vessel occlusion, analysis of previous *in vivo* experiments [16,47] has suggested that occlusion may be achieved by a complex cascade of events, possibly involving narrowing of the lumen by vascular spasm and radiation force, heating of the vessel wall to temperatures causing coagulation of collagen, edema of surrounding tissue, thermal and mechanical damage to the endothelium, activation and aggregation of platelets, and intraluminal thrombus formation. Feedback control could potentially allow occlusion to be achieved by allowing greater delivery of acoustic energy and thus increased heating of the vessel wall, while avoiding acoustic emissions predictive of vessel rupture.

Based on the results of the rupture prediction experiments performed here, which show the successful prediction of vessel rupture from measured subharmonic acoustic emissions, a real-time feedback control module was implemented and tested using a setup adapted from the prediction experiments. *Ex vivo* porcine superficial femoral arteries and subclavian arteries were subjected to 3.3-MHz HIFU controlled by this feedback module. HIFU intensity was modulated by the feedback control unit based on measured subharmonic emissions, using a threshold determined empirically from results of the rupture prediction experiments. This rupture suppression study had the major goal to determine whether passive detection of acoustic emissions, coupled with real-time feedback for HIFU intensity modulation, could delay vessel rupture, thus potentially improving the safety and efficacy of HIFU vessel occlusion.

## Materials and methods

### Rupture prediction experiments: experimental methods

**Figure 1:**
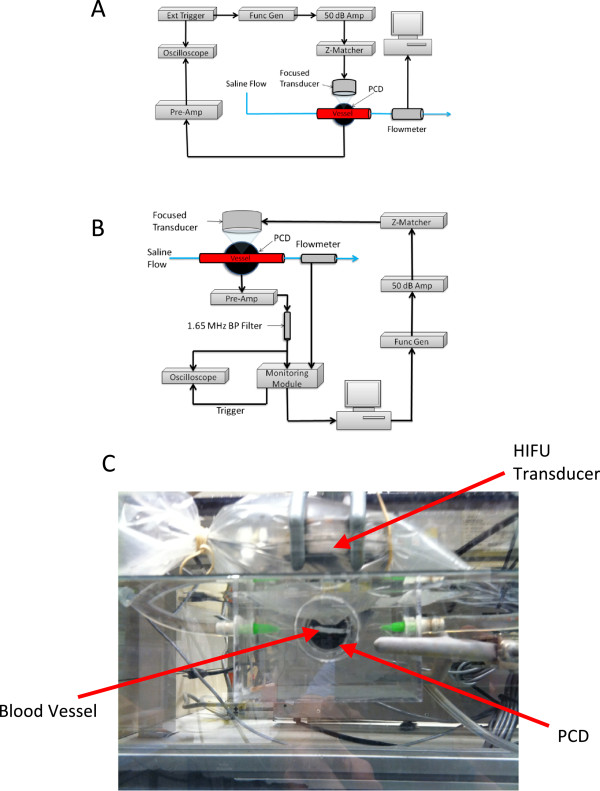
**Schematic showing experiment layout, experimental setup for rupture suppression, and Intra-procedural photograph showing positions of HIFU transducer. (A)** Schematic showing the experiment layout for rupture prediction trials. **(B)** Experimental setup for rupture suppression trials utilizing active feedback using a custom control module. **(C)** Intra-procedural photograph showing positions of the HIFU transducer, PCD, and *ex vivo* vessel.

The experiments reported here employed sonication of *ex vivo* porcine arteries by a 3.3-MHz HIFU transducer, with inline measurements of flow rate to detect rupture and a passive cavitation detector (PCD) to monitor bubble activity. An overview of the experimental setup is shown in Figure 1A. An open-loop fluid circuit was created with plastic tubing and a peristaltic pump. A 3-cm segment of porcine superficial femoral artery was interposed in the flow circuit by cannulation of 19-gauge needles into both ends. A peristaltic pump buffered by a closed air chamber created continuous flow of degassed saline through the circuit. The HIFU transducer was fixed over the vessel and the PCD was fixed at the level of the vessel, both perpendicular to the flow of saline.

Due to their ease of harvest and comparable size to potential targets of vessel occlusion (2–3 mm), the bilateral superficial femoral arteries of swine were harvested for this study. Necropsy was performed on two swine within 1 h postmortem. These animals were supplied and sacrificed by a different investigator under a protocol approved by the Institutional Animal Care and Use Committee (IACUC) of the University of Cincinnati. Branching vessels of the superficial femoral arteries were ligated with silk suture. The vessels were stored at 4**°**C in 0.9% saline solution with 2 g dextrose per liter. The sonications were performed within 48 h postmortem. The vessels were suspended in degassed 0.9% phosphate-buffered saline solution in an acrylic cuvet with degassed saline circulated by a peristaltic pump (Harvard Apparatus, Holliston, MA, USA) through the vessel at 63 mL/min during insonation. The peristaltic flow was buffered by allowing air compression via a T-connector to a closed syringe to smooth transient fluctuations in the flow velocity. The container with the treated vessel was placed into deionized water in a 10-gallon tank where an unfocused PCD (Panametrics NDT V302, 1 MHz, Billerica, MD, USA) was submerged (Figure 1C). The PCD was fixed in the same position relative to the vessel and HIFU transducer for all trials, ensuring that its sensitivity to HIFU-induced acoustic emissions was equivalent throughout all experiments.

Flow through the vessel was monitored with a flow meter (KOBOLD DPM Pelton wheel flow sensor, Pittsburgh, PA, USA) interfaced with a low-level direct current (DC) data logger (Madgetech Process101A, Warner, NH, USA), which sampled the flow-meter output current at 1 Hz. For an unruptured vessel, the flow-meter output current was found to fluctuate approximately ±1%. Detectable vessel rupture was thus defined as a 3% decrease in the flow-meter output current. In order to synchronize flow measurements with PCD-measured acoustic emissions, the timing of rupture detection was determined by measuring the delay between manual vessel puncture with a 23-gauge needle and 3% decrease in the flow-meter output. This delay was found to be 8 s within measurement precision, and thus, the instant of vessel rupture was defined as 8 s prior to detection of a 3% decrease in the flow-meter output.

The 3.3-MHz spherically focused transducer (Sonic Concepts, Bothell, WA, USA) used for insonation of the vessels (Figure 1) had a surface area of 34.6 cm^2^ and focal length of 62.6 mm, resulting in a focal width of 0.42 mm and a focal depth of 3.3 mm. A plastic cone attached to the transducer face enclosed the prefocal acoustic propagation path. The transducer and cone were suspended in deionized, degassed water within a latex sheath (Sheathing Technologies Inc., Morgan Hill, CA, USA), which was then placed within the cuvet containing the vessel in saline. The saturation of dissolved O_2_ of saline was measured and recorded before the start of insonations (range 13.5–19.5%). Vessel targeting was done by manual placement of the cone tip above the vessel. An ultrasonic pulser-receiver (Panametrics Model 5052UAX50, Billerica, MD, USA) was then used for pulse-echo ranging of the vessel relative to the HIFU transducer, with echo amplitude observed on a digital oscilloscope (LeCroy WaveRunner 6050A, Chestnut Ridge, NY, USA). The HIFU transducer was scanned using a two-axis stepping motor system (Velmex VXM 2, Bloomfield, NY, USA) to maximize echo amplitude, with the goal of precisely focusing the HIFU field on the proximal vessel wall, within the main sensitivity pattern of the unfocused PCD. For each ruptured vessel, a small puncture was observed at this focal location on the proximal vessel wall.

Insonations were performed at 3.3 MHz in 0.5-s intervals with 0.5-s pauses (1 Hz, 50% duty cycle) for up to 300 cycles (5 min) or until vessel rupture was detected. This pulsing scheme allowed the PCD signal from the entire 0.5-s duration of a HIFU pulse to be recorded before commencement of the next pulse, so that acoustic emission data was recorded whenever the vessel was subjected to HIFU. The time limit of 300 s was chosen to exceed durations of all previously published trials demonstrating successful vessel occlusion by HIFU, for which the maximum stated treatment duration was 180 s [16]. Waveforms were synthesized by a signal generator (Agilent 33220A, Santa Clara, CA, USA), triggered by a second function generator (Philips PH 5193, Seattle, WA, USA), amplified by a 50-dB radiofrequency power amplifier (ENI 3100 L, Kent, WA, USA), and transmitted to the HIFU transducer through an impedance matching network (Sonic Concepts, Bothell, WA, USA).

### Rupture prediction experiments: data processing

Acoustic emissions detected by the PCD were amplified by a low-noise preamplifier with amplitude gain 100 (Stanford Research Systems SR560, Sunnyvale, CA, USA). This preamplifier also performed high-pass filtering with a cutoff frequency of 10 kHz and slope of 12 dB/octave. The amplified and filtered PCD signals were then recorded by a digital oscilloscope (LeCroy WaveRunner 6050A, Chestnut Ridge, NY, USA) at a sampling rate of 10 MHz. PCD signals of length 5,000,000 samples were recorded for each 0.5-s sonication cycle throughout each sonication trial. PCD emissions were manually synchronized with the recorded flow data with 1-s precision.

To estimate the focal intensity incurred by the vessel, acoustic power measurements were taken using a radiation force balance with a cone reflector (Ohmic Instruments UPM-DT-10, Easton, MD, USA). Acoustic power was measured for the input range 50–350 mVpp at 50-mVpp intervals, amplified by the same radiofrequency amplifier used in the sonication trials. Acoustic power for intermediate input voltages was estimated by linear interpolation. For the range of sonication amplitudes employed in the trials described below, measured acoustic power ranged from 20.28–32.75 W. These measured acoustic power readings were used as inputs to a simulation employing the KZK equation [48], along with the appropriate transducer dimensions and assuming water as the propagation medium, to estimate the corresponding spatial-peak, temporal-peak (SPTP) focal intensities as 15,690–24,430 W/cm^2^, corresponding to peak negative pressures of 10.92–12.09 MPa (HIFU Simulator v1.2 by Joshua Soneson, US Food and Drug Administration; MATLAB R2011a, The MathWorks, Natick, MA, USA).

HIFU exposure conditions for the 18 trials reported here are shown in Table 1. Trial sonications at lower intensities (three trials with estimated SPTP focal intensity 12,429 W/cm^2^) resulted in no vessel ruptures and were thus excluded from analysis.

**Table 1:** Exposure conditions and times to rupture for all vessels sonicated in the rupture prediction experiments

**Trial**	**AWG output voltage (mVpp)**	**Measured acoustic power (W)**	**Computed focal intensity I**_**SPTP **_**(W/cm**^**2**^**)**	**Computed peak negative pressure (MPa)**	**Time to rupture (s)**
1	250	20.28	15,690	10.92	29
2	250	20.28	15,690	10.92	133
3	275	26.45	20,570	11.83	27
4	275	26.45	20,570	11.83	25
5	275	26.45	20,570	11.83	NR
6	275	26.45	20,570	11.83	NR
7	280	26.96	20,940	11.90	NR
8	285	27.53	21,350	11.97	14
9	285	27.53	21,350	11.97	32
10	300	28.42	21,980	12.09	55
11	300	28.42	21,980	12.09	41
12	300	28.42	21,980	12.09	60
13	300	28.42	21,980	12.09	19
14	300	28.42	21,980	12.09	133
15	300	28.42	21,980	12.09	35
16	325	32.06	24,430	12.52	55
17	325	32.06	24,430	12.52	67
18	325	32.06	24,430	12.52	42

In trials 5–7, NR indicates that no rupture occurred. *AWG* arbitrary waveform generator.

Acoustic emissions were quantified by spectral analysis of the PCD signals using custom software written in MATLAB. Each 5,000,000-point, 0.5-s PCD signal was split into 1,000 segments of length 5,000 samples with no overlap. To estimate power spectra for each 0.5-s PCD signal, the discrete Fourier transform of each segment was computed and the squared magnitudes of the 1,000 individual segments were averaged. The resulting frequency resolution was 10 kHz. To quantify the electronic noise background, reference PCD signals for each trial were obtained prior to insonation, with all equipment powered on but the function generator, and these reference noise signals were processed in the same manner.

In order to differentiate between the various types of bubble activity, measured power spectra were integrated over specific frequency bands, similar to a previous study [25]. The integrated signal power was then divided by the integrated power of the reference noise signal for the same band, resulting in a computed time-dependent signal-to-noise ratio (SNR) for each emission type. Emission energy within a low-frequency (10–30 kHz) band was associated with tissue or fluid vaporization and boiling [26,27]. To characterize broadband emissions associated with inertial cavitation, measured power spectra were integrated from 0.3–1.1 MHz [25]. Subharmonic emission energy from the single bin at 1.65 MHz was also quantified as an indicator of stable cavitation [40,41]. Due to the nonuniform frequency response of the PCD, comparisons between low-frequency, broadband, and subharmonic emissions could not be performed.

PCD signal energy within each of these bands was computed for each 0.5-s sonication interval, yielding time-dependent low-frequency, broadband, and subharmonic signal levels with 1-s temporal resolution. In order to test the potential of passive cavitation detection to predict vessel rupture, individual PCD signal spectra were classified either as ‘pre-rupture’ emissions (0–10 s prior to the estimated instant of rupture) or ‘intact-vessel’ emissions (more than 10 s prior to the instant of rupture). The duration of 10 s for the pre-rupture period was chosen, based on observation of measured acoustic emissions, to capture the main temporal variations in emission levels, while smoothing out short-lived statistical fluctuations. The effect of window length was assessed in a *post hoc* study, in which receiver operating characteristic curves were computed for window lengths of 1–20 s.

Statistical analysis included comparison of low-frequency, broadband, and subharmonic emission levels between pre-rupture and intact-vessel emissions as well as receiver operating characteristic (ROC) curve analysis to test prediction of vessel rupture based on each emission level. These analyses were performed using custom software written in MATLAB. Primary analysis included all measured acoustic emissions, up to the time of vessel rupture or the 5-min time limit, from all 18 trials.

Two-tailed, independent-sample *t* tests were performed comparing intact-vessel emissions to pre-rupture emissions, with assumption of equal but unknown variance. In addition, means and standard errors of the mean (SEM) were computed for the measured energy of pre-rupture and nonrupture low-frequency, broadband, and subharmonic emissions. These analyses were performed for all 18 trials, with the significance criterion *p* < 0.05.

Receiver operating characteristic (ROC) curves, which plot sensitivity and specificity of a binary classifier as a function of a predictive parameter (test variable) threshold, were computed to assess the utility of passive cavitation detection for predicting vessel rupture. In this analysis, carried out separately for measured low-frequency, broadband, and subharmonic emissions, test variables were the measured emission levels for each 0.5-s PCD signal, while state variables were the classification of each PCD signal as a nonrupture or pre-rupture emission. Plotting the true-positive rate (sensitivity) vs. the false-positive rate (1-specificity) results in an ROC curve. Area under the ROC curve is an indicator of prediction success, with area 1 indicating a perfect predictor and area 0.5 indicating performance no better than chance. Statistical significance of each predictor was assessed vs. the null hypothesis of area 0.5, with the significance criterion *p* < 0.05.

### Rupture suppression experiments

Using the data obtained from the vessel rupture prediction study, a control module was designed to actively monitor the HIFU treatment and adjust ultrasound intensity in an attempt to maximize acoustic power supplied to the treated vessels while avoiding rupture. Since subharmonic emissions were found most predictive of vessel rupture, measured subharmonic emissions were used as input to the control module, which modulated the sonication amplitude to avoid acoustic cavitation. The setup for these experiments, similar to the rupture prediction experiments except for the adjustments in HIFU exposure levels, is shown in Figure 1B.

**Figure 2:**
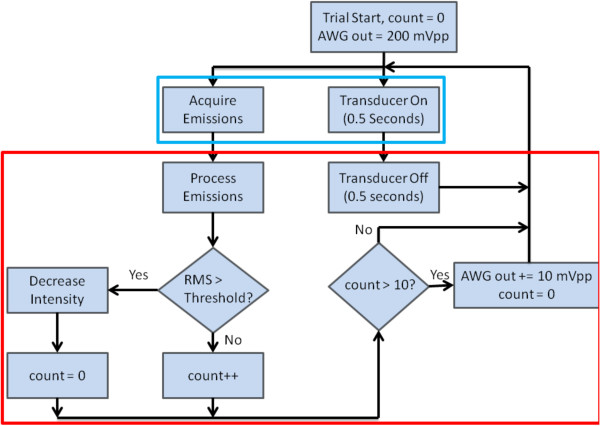
**Flow chart detailing the vessel rupture suppression protocol.** Subharmonic emissions are acquired while the HIFU is active. During the time the transducer is off, the emissions are processed and a calculated value compared to a given threshold. If this value surpasses the threshold, the ultrasound intensity is decreased. If the threshold is not reached for ten consecutive counts (10 s), the intensity is increased (up to a maximum of 24,430 W/cm^2^). This process is repeated for a total of 300 cycles.

In rupture suppression trials, porcine subclavian and bilateral superficial femoral arteries were exposed to focused ultrasound as in the above rupture prediction experiments, starting with a 9,117 W/cm^2^ SPTP focal intensity and increased stepwise by the controller to a maximum of 21,980 W/cm^2^ SPTP. Trials were deemed either ‘controller-active’ or ‘controller-inactive.’ In controller-active trials, a monitoring module decreased the sonication amplitude whenever measured subharmonic emissions exceeded a pre-defined threshold, defined below. A flow diagram for the controller actions is detailed in Figure 2. For comparison, paired controller-inactive trials, with the feedback controller disabled, were performed on vessel segments from the same length of artery on the same day. As for the rupture prediction experiments, sonications were limited to 300 s, a time exceeding that required for HIFU vessel occlusion in previously published studies [16].

Emissions recorded by the PCD were filtered by a 1.65-MHz bandpass filter before amplification by the same pre-amplifier employed in the rupture prediction experiments (gain 20,000) and sent to the monitoring module, in which the signal was half-wave rectified, passed through a 1,063-Hz AM demodulator, and converted to a digital signal by the onboard microcontroller at a sampling rate of 68.6 kHz. RMS subharmonic emission values were computed from these signal-envelope samples for each 0.5-s insonation, resulting in time-dependent subharmonic emission recordings with a temporal resolution of 1 s. Emissions were recorded by the oscilloscope for verification, but were not used in postprocessing.

The subharmonic threshold value used by the controller module was determined by simulating the effects of the feedback-controller signal chain on PCD signals previously measured in the rupture prediction experiments. Transfer functions of the preamplifier, 1.65-MHz bandpass filter, and half-wave rectifier/AM demodulator combination were modeled in MATLAB, and previously measured PCD signals were subjected to these transfer functions in the given order. Receiver operating characteristic curves were then generated in the same manner as for the rupture prediction experiments, and the point on the ROC curve simultaneously maximizing sensitivity and specificity (i.e., the point closest to the corner [0,1]) was determined. To obtain a more conservative threshold appropriate for avoidance of vessel rupture, the measured RMS noise level was then subtracted 1.5 times from the threshold simultaneously maximizing sensitivity and specificity. For this adjusted threshold, found to be 0.06 V_RMS_, the simulated sensitivity based on data from the rupture prediction study was 0.606; specificity was 0.702; positive predictive value (PPV) was 0.188; and negative predictive value (NPV) was 0.939.

Statistical analysis was performed to compare time to rupture for the controller-active and controller-inactive groups, the trial-average ultrasound intensity, and the total acoustic energy delivered. Analysis included all measured subharmonic emissions and intensities, up to the point of rupture, for all experiment pairs. If a treated vessel did not rupture after the 300-s exposure, the rupture time was conservatively defined as 300 s for these analyses. A one-sided paired Wilcoxon signed-rank test, which compares the population mean ranks of two samples, was utilized to determine if the controller-active trials had a longer time to vessel rupture than the controller-inactive cases. Single-sided, paired *t* tests were performed to compare the time-averaged, spatial-peak-temporal peak focal intensity and the total acoustic energy delivered during the controller-active and controller-inactive trials, testing the hypotheses that the controller allowed delivery of greater time-averaged focal intensity and greater total acoustic energy. The criterion for statistical significance in all statistical tests was *p* < 0.05.

## Results

### Rupture prediction experiments

Of the 18 vessels sonicated in the rupture prediction experiments, 15 vessels ruptured prior to 300 s of sonication, while 3 vessels were sonicated for 300 s without rupture. Times to rupture, together with HIFU exposure conditions for each trial, are shown in Table 1. SPTP focal intensities varied from 15,690–24,430 W/cm^2^. No rupture occurred for two trials at SPTP 20,570 W/cm^2^ and one trial at 20,940 W/cm^2^. For the 15 vessels that ruptured, the mean sonication time to rupture was 51 s (range = 14–133 s, standard deviation = 37 s). For the 15 ruptured vessels, time to rupture was not significantly correlated with focal intensity (*r* = -0.1814, *p* = 0.5177).

**Figure 3:**
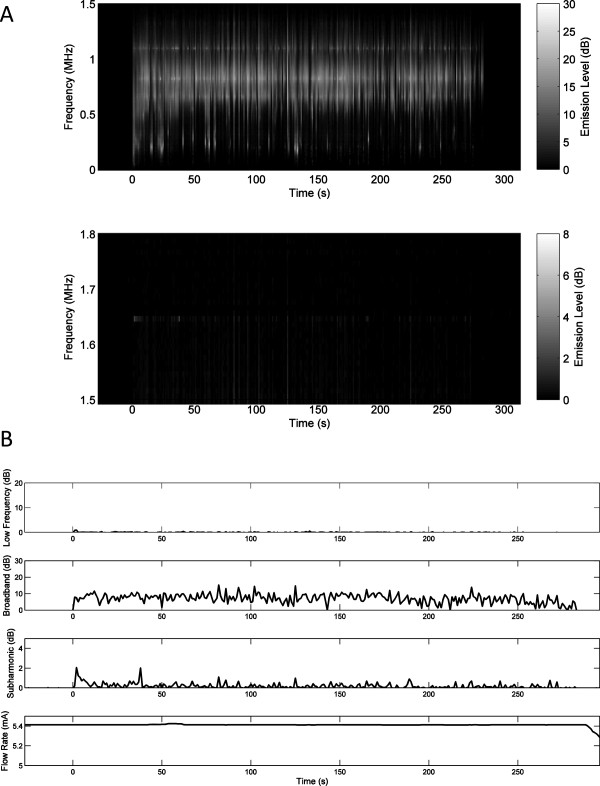
**Spectrogram of time-dependent passive cavitation detector signals and plots of time-dependent acoustic emissions for trial 5. (A)** Spectrogram of time-dependent passive cavitation detector signals (in dB-scaled SNR) for trial 5 with 20,570 W/cm^2^ SPTP focal intensity. **(B)** Plots of time-dependent acoustic emissions (dB-scaled SNR) and flow-meter output current for trial 5. No vessel rupture occurred in this trial.

**Figure 4:**
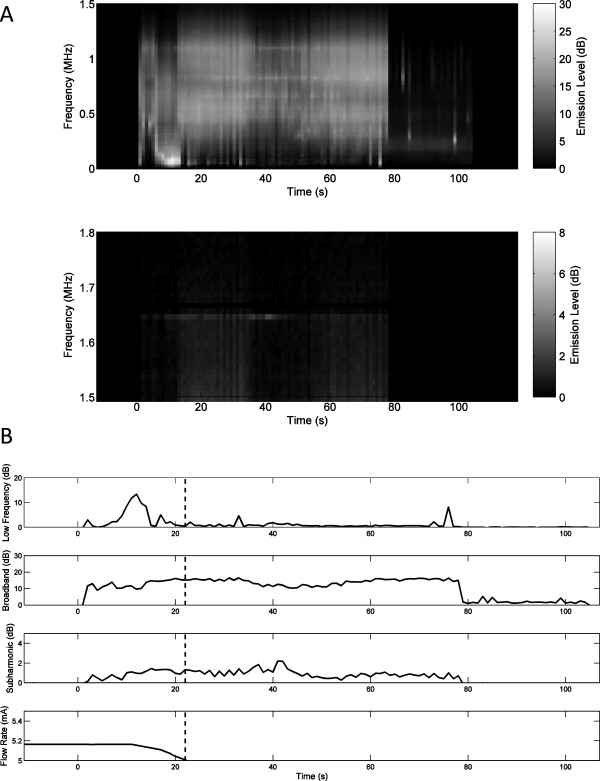
**Spectrogram of time-dependent passive cavitation detector signals and time-dependent acoustic emissions for trial 8. (A)** Spectrogram of time-dependent passive cavitation detector signals (dB-scaled SNR) for trial 8 with 21,350 W/cm^2^ SPTP focal intensity. **(B)** Time-dependent acoustic emissions (dB-scaled SNR) and flow-meter output current for trial 8. The estimated time of vessel rupture is shown by the dashed vertical line.

**Figure 5:**
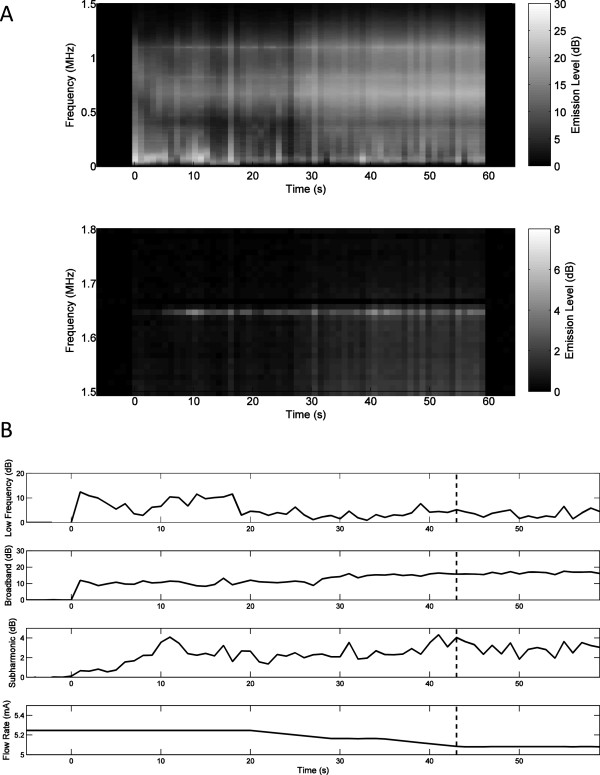
**Spectrogram of time-dependent passive cavitation detector signals and time-dependent acoustic emissions for trial 15. (A)** Spectrogram of time-dependent passive cavitation detector signals (in dB SNR) for trial 15 with 21,980 W/cm^2^ SPTP focal intensity. **(B)** Time-dependent acoustic emissions (in dB-scaled SNR) flow-meter output current for trial 15. The estimated time of vessel rupture is shown by the dashed vertical line.

Representative plots of measured low-frequency, broadband, and subharmonic acoustic emissions during insonation are displayed as decibel-scaled SNR levels in Figures 3, 4, and 5. Figures 3A, 4A, and 5A show measured PCD power spectra as a function of time and frequency for trials 5 (20,570 W/cm^2^, no rupture), 8 (21,350 W/cm^2^, rupture at 14 s), and 15 (21,980 W/cm^2^, rupture at 35 s). Spectrograms are shown for two frequency ranges, 0–1.5 MHz (including low-frequency and broadband emissions) and 1.5–1.8 MHz (including subharmonic emissions), with different dynamic ranges chosen to distinctly display emissions within both bands. Figures 3B, 4B, and 5B show the corresponding measured time-dependent low-frequency, broadband, and subharmonic emission levels for the same trials, together with the synchronously measured flow-meter output current. Broadband emissions in all three of these cases are measured at mean levels >5 dB SNR and are slightly higher in the trials resulting in vessel rupture. Low-frequency and subharmonic emissions are substantially larger in trials 8 and 15, with vessel rupture (Figures 4 and 5), compared to trial 5, without vessel rupture (Figure 3).

Qualitative assessment of time-dependent emissions levels from all trials showed several common trends. In many cases, low-frequency, broadband, or subharmonic levels were elevated immediately after initiation of sonication, then markedly declined within 10–20 s. These higher initial levels were likely caused by residual gas bubbles, which were then eliminated by the high-intensity sonication. Relative initial levels for the three emission types varied between trials, but were generally highest for low-frequency emissions and smallest for broadband emissions. For example, in Figure 3B, the subharmonic emission level is elevated immediately after sonication begins, then declines to near baseline levels within about 10 s. Similarly, in Figure 5B, low-frequency emission levels at the start of sonication are elevated to near the maximum level achieved later in the exposure.Subharmonic emissions were generally lower for nonrupture trials. The highest subharmonic emission for any nonrupture trial occurred immediately after commencement of sonication in trial 5 (Figure 3). Figure 4B shows typical trends for the trials resulting in vessel rupture. After the initial burst, emission levels in each frequency band steadily increase over time until the vessel ruptures. However, in some cases, including that shown in Figure 5B, low-frequency and broadband emissions did not increase steadily, and no clear relation between vessel rupture and the time-dependent emission levels was evident.

**Figure 6:**
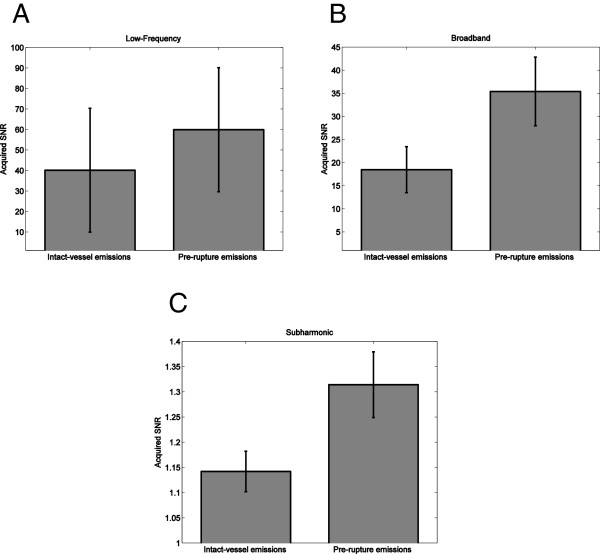
**Means and standard errors of intact-vessel and pre-rupture emission levels for all trials (*N*** **= 18). (A)** Low-frequency levels. **(B)** Broadband levels. **(C)** Subharmonic levels.

Statistical comparison of pre-rupture emissions (all emissions within 10 s prior to rupture) to intact-vessel emissions (all emissions greater than 10 s before rupture and all emissions from the nonruptured trials) were performed based on the measured signal energy of each emission type from each 0.5-s PCD signal. Averaged pre-rupture emission level energies, quantified as decibel-scaled SNR, were 17.71, 15.49, and 1.18 dB for the low-frequency, broadband and subharmonic frequency bands, respectively. Corresponding levels were 16.03, 12.67, and 0.58 dB for intact-vessel emissions. The mean and standard error of each emission level for pre-rupture and intact-vessel emissions, as SNR without logarithmic scaling, are shown in Figure 6. An independent-sample *t* test, comparing intact-vessel emissions to pre-rupture emissions with assumption of equal variance, indicates significantly higher broadband (*p* < 10^-19^, *t* = 9.38, *N* = 18) and subharmonic (*p* < 10^-29^, *t* = 11.64, *N* = 18) levels for pre-rupture emissions. Low-frequency levels for pre-rupture emissions were also higher than for intact-vessel emissions, but this difference was not statistically significant (*p* = 0.0582, *t* = 0.896, *N* = 18).

**Figure 7:**
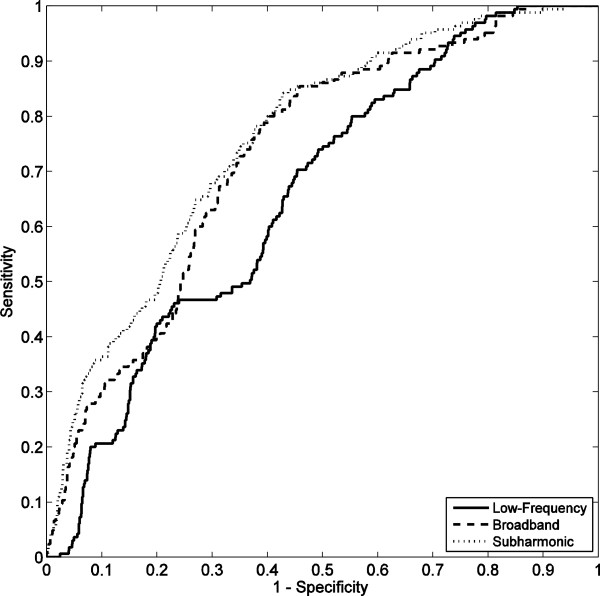
**ROC curves for prediction of vessel rupture, based on 18 trials.** Curves are shown quantifying rupture prediction performance based on low-frequency, broadband, and subharmonic emissions within 10 s before rupture.

ROC analysis on data from all 18 trials, illustrated in Figure 7, indicated that passive cavitation detection of low-frequency, broadband, and subharmonic emissions was predictive of vessel rupture for all three emission types. Areas under the ROC curve (AUROC) values were 0.657 (*p* < 10^-11^), 0.729 (*p* < 10^-16^), and 0.757 (*p* < 10^-16^) for low-frequency, broadband, and subharmonic emissions, respectively. These AUROC values are shown with corresponding standard errors and confidence intervals in Table 2.

**Table 2:** AUROC statistics for vessel rupture prediction by low-frequency, broadband, and subharmonic emissions

**Acoustic emission type**	**AUROC**	**Standard error**	***p *** **value**	**95% confidence interval**	
				**Lower bound**	**Upper bound**
Low-frequency	0.657	0.024	<10^-11^	0.610	0.704
Broadband	0.729	0.023	<10^-16^	0.684	0.774
Subharmonic	0.757	0.022	<10^-16^	0.713	0.780

Areas under the ROC curves (AUROC) with corresponding standard errors (under the nonparametric assumption), asymptotic *p*-values relative to the null hypothesis AUROC = 0.5, and 95% confidence intervals (18 trials).

Threshold values for each emission type to maximize true-positive rate (sensitivity) and true-negative rate (specificity) were determined using data from all 18 trials. These thresholds are given as decibel-scaled SNR values, together with the resulting measured sensitivities, specificities, positive predictive values, and negative predictive values, in Table 3. Subharmonic emissions were most predictive of vessel rupture. For the subharmonic threshold, the sensitivity was 74.5%; the specificity was 64.4%; the positive predictive value was 19.0%; and the negative predictive value was 95.7%.

**Table 3:** Optimal emission thresholds and corresponding accuracy metrics for rupture prediction

**Acoustic emission type**	**Threshold (dB)**	**Sensitivity**	**Specificity**	**PPV**	**NPV**
Low-frequency	0.863	69.6%	54.7%	14.7%	94.1%
Broadband	11.04	75.1%	62.9%	18.5%	95.7%
Subharmonic	0.499	74.5%	64.4%	19.0%	95.7%

Threshold values (in dB relative to the measured noise floor) yielding maximum sensitivity and specificity, with corresponding sensitivities, specificities, positive predictive values (PPV), and negative predictive values (NPV), using data from 18 trials.

In a *post hoc* study to determine the effect of the pre-rupture period length, ROC curves were computed for subharmonic emissions using pre-rupture periods of duration 1–20 s. With this range of window lengths, areas under the ROC curve (AUROC) were found to range from a minimum of 0.746 to a maximum of 0.781, varying by no more than 3.1% from the AUROC for the chosen window length of 10 s. Thus, prediction efficacy was determined not to depend strongly on the choice of duration for the pre-rupture emission period.

### Rupture suppression experiments

**Figure 8:**
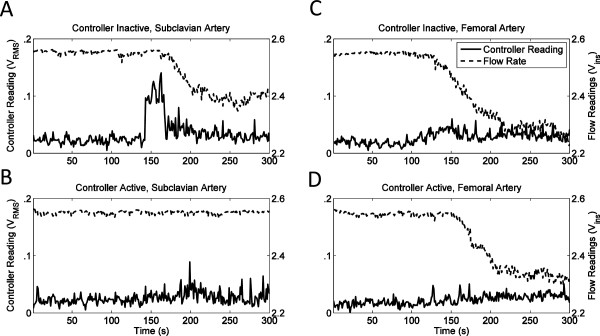
**Subharmonic emissions and flow rate recorded by monitoring module during rupture suppression experiments. (A)** Subclavian artery, controller inactive, pair 4. **(B)** Subclavian artery, controller active, pair 4, subharmonic detection threshold set to 0.06. **(C)** Femoral artery, controller inactive, pair 5. **(D)** Femoral artery, controller active, pair 5, subharmonic detection threshold set to 0.06.

Representative plots of subharmonic emissions and flow-meter output during the rupture suppression experiments, as recorded by the control module, are shown in Figure 8. Figure 8A,B (vessel pair 4) shows typical trends observed for the seven subclavian arteries employed pairs in these experiments. In the controller-inactive trials, subharmonic emissions typically increased greatly later in the exposures (after about 150 s of sonication) and then remained at an elevated level, followed by a vessel rupture shortly thereafter. In the controller-active trials, increased subharmonic emissions typically appeared as brief spikes that were not sustained after the HIFU sonication level was reduced by the controller. Figure 8C,D (vessel pair 5) shows typical trends for the three femoral arteries in these experiments. For femoral arteries in both the controller-active and controller-inactive groups, subharmonic emissions showed smaller amplitude spikes and did not show sustained elevations in the emission levels.

**Figure 9:**
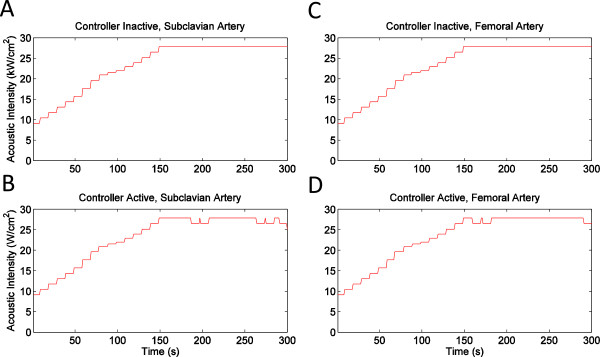
**Ultrasound intensity at the focus versus time for representative trials in the vessel rupture suppression experiments. (A)** Subclavian artery, controller inactive, pair 4. **(B)** Subclavian artery, controller active, pair 4, subharmonic detection threshold set to 0.06. **(C)** Femoral artery, controller inactive, pair 5. **(D)** Femoral artery, controller active, pair 5, subharmonic detection threshold set to 0.06.

Figure 9 shows plots of the HIFU time-dependent SPTP focal intensity for the same trials depicted in Figure 8. Figure 9A,C shows a monotonic increase in intensity, as specified for all controller-inactive trials. For the controller-active trials illustrated in Figure 9B,D, decreases in focal intensity occurred after the 0.06-V_RMS_ subharmonic emission threshold was reached, triggering a 10-mVpp decrease in the output of the function generator. In two cases (vessel pairs 8 and 10), the subharmonic emission threshold was never reached during the controller-active trials.

**Figure 10:**
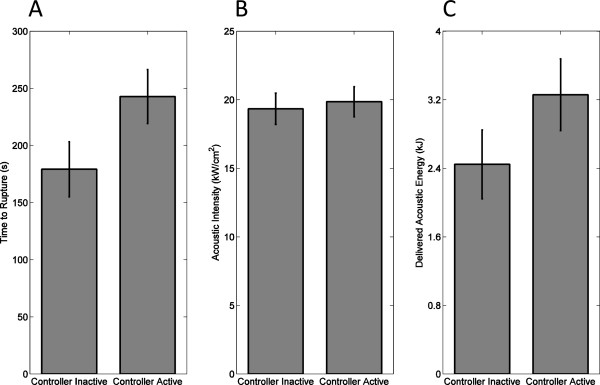
**Comparison of controller-inactive and controller-active trials in rupture suppression experiments.** Means and standard errors are shown for **(A)** time to rupture from start of sonication, **(B)** trial-average SPTP acoustic intensity at the focus, and **(C)** total acoustic energy delivered.

Rupture occurrence and timing in the rupture suppression experiments is summarized in Table 4. Statistical comparison using a one-sided, paired Wilcoxon signed-rank test indicated that controller activation caused a significant delay in vessel rupture (*T*_-_ = 8, *p* = 0.0244, *N* = 10). Times to rupture (mean ± standard deviation) were 242.8 ± 74.7 s for the controller-active group and 179.1 ± 76.1 s for the controller-inactive group, as illustrated in Figure 10A. For the ten pairs of vessels tested, the controller-active vessel had a longer time to rupture in seven cases; the controller-inactive vessel had a longer time to rupture in two cases; and neither vessel ruptured in one case. Nine of ten vessels within the controller-inactive group ruptured within the 300-s time limit (minimum time to rupture 22 s), including three of three femoral arteries and six of seven subclavian arteries. Within the controller-active group, four vessels ruptured within the 300-s time limit (minimum time to rupture 131 s), including all three femoral arteries and one subclavian artery.

**Table 4:** Time to rupture from experiment start for all vessel rupture suppression trials

**Pair number**	**Time to rupture, controller inactive (s)**	**Time to rupture, controller active (s)**	**Pair difference (s)**	**Vessel type**
1	282	300	18	Subclavian
2	170	300	130	Subclavian
3	151	131	-20	Femoral
4	180	300	120	Subclavian
5	144	167	23	Femoral
6	189	155	-34	Subclavian
7	173	300	127	Subclavian
8	22	175	153	Femoral
9	300	300	0	Subclavian
10	180	300	120	Subclavian

The values listed in the ‘Pair difference’ column correspond to those used in the paired Wilcoxon signed-rank test and the paired *t* test. A time to rupture of 300 s indicates that no rupture occurred during the experiment.

Qualitatively, femoral arteries were found to rupture sooner than subclavian arteries in these experiments, possibly due to their smaller wall thickness. However, in the absence of feedback control, the fraction of vessels rupturing was not significantly different for the two vessel types (Fisher’s exact test, *p* = 1.0). In the presence of feedback control, the fraction of femoral vessels rupturing within the 300-s sonication period was significantly greater than the fraction of subclavian vessels rupturing (Fisher’s exact test, *p* = 0.0333). However, the delay in vessel rupture between controller-active and controller-inactive states was not significantly different when comparing subclavian vs. femoral vessels (Student *t* test, *p* = 0.759).

Statistics of average focal intensity and delivered acoustic energy for the controller-active and controller-inactive groups are illustrated in Figure 10B,C. The mean HIFU SPTP focal intensities throughout the controller-active and controller-inactive trials were comparable, as shown in Figure 10B. The trial-mean focal intensity was 19,852 ± 3,461 W/cm^2^ (mean ± standard deviation, *N* = 10) for the controller-active trials and 19,335 ± 3,593 W/cm^2^ for the controller-inactive trials, a statistically insignificant difference (one-sided, paired *t* test, *t* = 0.362, *p* = 0.363, *N* = 10). As shown in Figure 10C, the average acoustic energy delivered was greater for controller-active trials (mean ± standard deviation 3,259 ± 1,323 J, *N* = 10) than for controller-inactive trials (2,446 ± 1,267 J, *N* = 10), a statistically significant difference (one-sided, paired *t* test, *t* = 2.037, *p* = 0.0361, *N* = 10).

## Discussion

In these trials applying HIFU to *ex vivo* porcine subclavian and superficial femoral arteries, passive cavitation detection results suggest that acoustic emissions can be used to predict imminent vessel rupture and as a feedback mechanism to suppress vessel rupture. In the rupture prediction experiments reported here, significant increases were observed in both subharmonic emission levels (*p* < 10^-29^) and broadband emission levels (*p* < 10^-19^) for pre-rupture emissions (within 10 s immediately prior to vessel rupture) compared to intact-vessel emissions as defined above. Notably, subharmonic emissions were found to predict vessel rupture more effectively than broadband emissions (AUROC 0.757 vs. 0.729), despite the lower signal-to-noise ratio for subharmonic emissions in these experiments, illustrated in Figures 3, 4, and 5. Subharmonic emissions predicted vessel rupture with a sensitivity of 74.5%, specificity of 64.4%, and a negative predictive value of 95.7%.Results of the present rupture prediction study suggest that sustained acoustic cavitation activity, including both stable and inertial cavitation, is associated with vessel rupture during HIFU application. As seen in Figures 3, 4, and 5, acoustic emission levels tended to remain relatively steady or increase with time. Since acoustic cavitation could only occur during the 0.5-s HIFU pulses and not during the alternating 0.5-s rest periods, this suggests that cavitation could be nucleated similarly within each sonication pulse. Nucleation likely did not occur within the vessel lumen, because the lumen contained only degassed saline circulating in an open-loop flow path. Similarly, the exterior of the vessel was surrounded by degassed saline. Thus, bubble nucleation and acoustic cavitation were likely isolated within the vessel wall. HIFU-induced damage to the vessel wall, potentially caused both by heating and by the mechanical effects of cavitation, may have facilitated sustained bubble nucleation, resulting in growing cavitation with increasing exposure time. Results of both the rupture prediction and suppression studies suggest that the likelihood of vessel rupture increases as this cavitation activity grows.

Although the present studies show a significant link between acoustic cavitation and vessel rupture, they do not show that cavitation is the sole causative mechanism for rupture. In addition to direct mechanical effects of acoustic cavitation within the vessel wall, vessel heating likely plays a role [16]. In the present rupture prediction study, although low-frequency pre-rupture emissions were not significantly greater than intact-vessel emissions, low-frequency emissions (associated with tissue or fluid boiling) were significant predictors of vessel rupture, as shown in the ROC analysis. Low-frequency emissions were less effective predictors than either subharmonic or broadband emissions. These results suggest that heating-induced tissue vaporization, associated with low-frequency emissions, was not the primary cause of HIFU-induced vessel rupture in these experiments. However, tissue heating likely contributes to vessel rupture, by mechanisms including mechanical tissue damage caused directly by boiling [24] and indirectly by coagulative necrosis, potentially weakening tissue structures and enabling further mechanical damage from acoustic radiation force, streaming, and mechanical effects of cavitation.

Therapeutic tissue heating (temperature elevations >60°C) likely plays a greater role in coagulative occlusion of treated blood vessels. Threshold analysis of previous experiments on *in vivo* vessel coagulation [16] suggests that vessel coagulation is more likely at higher HIFU heating rates, consistent with the causation of occlusion by heating rather than acoustic cavitation. Possible mechanisms for vessel occlusion, supported by analysis of previous *in vivo* occlusion studies, have suggested a complex cascade of mechanisms for occlusion, with heating playing a major role through shrinkage and denaturation of collagen, elastin fragmentation and refusion, thermal damage to the endothelium, and tissue fusion within the vessel wall [16,47]. The lumen is narrowed by these thermal effects as well as smooth muscle contraction, vasospasm, and acoustic radiation force. These combined effects result in endothelial disruption activating platelets and the coagulation cascade and direct activation of platelets, intraluminal thrombus formation, and potentially fibrosis resulting in permanent occlusion [16,23,47,49].

For safe and effective application of HIFU for vascular occlusion, further refinement in treatment methods is needed to avoid vessel hemorrhage. The possible technique tested here was modulation of HIFU intensity through a feedback mechanism to reduce cavitation activity. This feedback loop triggered a reduction of the HIFU intensity after passively detected subharmonic emissions exceeded an empirical threshold determined using results of the rupture prediction study. This rupture suppression study showed that vessel rupture can be significantly delayed by feedback control of HIFU sonication amplitude based on measured subharmonic emissions (minimum 22 s, mean 179 s, standard deviation 76 s for the controller-inactive group; minimum 131 s, mean 243 s for the controller-active group; *p* < 0.03). The feedback controller caused a statistically insignificant difference in the time-averaged focal intensity delivered to the vessel, but allowed significantly more total acoustic energy to be delivered (*p* < 0.04), indicating that rupture suppression was associated with reducing cavitation activity rather than reducing the average HIFU exposure level. Thus, feedback control may potentially enable greater vessel heating while reducing mechanical damage associated with cavitation.

Although the feedback controller did not cause rupture to be avoided in all cases (4/10 vessels ruptured in the controller-active group vs. 9/10 for the controller-inactive group), the significantly increased time to vessel rupture may in practice allow vessel occlusion to be achieved before rupture. In our *ex vivo* model, vessel occlusion was not possible due to the lack of flowing blood and a living vessel wall, so that the coagulation cascade could not be initiated. For this reason, HIFU exposures continued well beyond durations of interest for vessel occlusion, up to a maximum of 300 s. In comparison, as summarized in ref. [6], previous studies demonstrating vessel occlusion by focused ultrasound have employed treatment durations of 180 s or less, with the great majority employing exposures less than 10 s. Thus, when used during vessel occlusion procedures, the present feedback control method could potentially achieve clinically valuable suppression of vessel rupture, substantially reducing the likelihood of hemorrhage during HIFU vessel occlusion, while allowing maximum delivery of HIFU energy for optimal thermal coagulation of the vessel.

Clinical application of this feedback control method to vessel occlusion will require further refinement and validation. Passive cavitation detection will need to be effectively implemented for the geometries of interest in particular applications, including neurosurgical [16], obstetric [47], and others. Optimal frequency bands for analysis of acoustic emissions (for example, choice of subharmonic or ultraharmonic bands to detect stable cavitation activity) will likely depend on the HIFU frequency and tissue path in the application of interest. Spatial selectivity in cavitation detection, achievable by passive cavitation imaging [44,50-52], may be useful, particularly in combination with pulse-echo methods for real-time detection of local heating, such as echo decorrelation imaging [53,54]. Optimal thresholds for emission detection would need to be determined more rigorously, with the goal of reducing hemorrhage likelihood to clinically acceptable levels. Thresholds would need to be determined separately based on factors including size and type of vessel, HIFU frequency, anatomical environment, and acceptable level of risk for the clinical application. The feedback control method will then need to be validated in conjunction with vessel occlusion in clinically realistic applications, testing its potential to heat of the vessel in a safer, more controllable manner to improve treatment efficacy while avoiding hemorrhage. Achievement of safe and consistent occlusion by this technique would show feasibility for this technology’s future role in neurosurgical applications [16].

## Conclusion

The experiments reported here indicate the important role of acoustic cavitation in HIFU-induced vessel rupture. In the rupture prediction experiments, subharmonic and broadband emissions were predictive of HIFU-induced vessel rupture, with subharmonic emissions providing greater predictive accuracy. This indicates that acoustic cavitation, particularly sustained cavitation accompanied by subharmonic emissions, may play a central role in vessel hemorrhage during HIFU application for vessel occlusion. Low-frequency emissions were less predictive of vessel rupture, suggesting that heating-induced tissue vaporization is not a primary mechanism of HIFU-induced vessel rupture. Rupture suppression experiments, implementing a real-time feedback controller to reduce acoustic cavitation by modulating HIFU intensity, show the potential to avoid vessel rupture during HIFU treatments while still delivering significant acoustic energy. Future investigations on vascular occlusion with HIFU may benefit from passive monitoring and control of subharmonic and broadband emissions to prevent hemorrhagic complications.

## Competing interests

The authors declare that they have no competing interests.

## Authors' contributions

Data acquisition and analysis detailed in this study were carried out by CLH and JCS, with the feedback control module designed and built by CLH. The initial draft of the manuscript was prepared by JCS and CLH, with revisions performed by TDM, CLH, MTB, and MZ. The study was conceived by TDM and initiated through work by MTB. All authors read and approved the final manuscript.
